# Residential Segregation and County-Level COVID-19 Booster Coverage in the Deep South: Surveillance Report and Ecological Study

**DOI:** 10.2196/44257

**Published:** 2023-12-05

**Authors:** Chengbo Zeng, Jiajia Zhang, Zhenlong Li, Xiaowen Sun, Huan Ning, Xueying Yang, Sharon Weissman, Bankole Olatosi, Xiaoming Li

**Affiliations:** 1 Department of Health Promotion, Education, and Behavior Arnold School of Public Health University of South Carolina Columbia, SC United States; 2 South Carolina SmartState Center for Healthcare Quality University of South Carolina Columbia, SC United States; 3 Big Data Health Science Center University of South Carolina Columbia, SC United States; 4 Department of Epidemiology and Biostatistics Arnold School of Public Health University of South Carolina Columbia, SC United States; 5 Geoinformation and Big Data Research Laboratory Department of Geography, College of Arts and Sciences University of South Carolina Columbia, SC United States; 6 Department of Internal Medicine School of Medicine University of South Carolina Columbia, SC United States; 7 Department of Health Services Policy and Management Arnold School of Public Health University of South Carolina Columbia, SC United States

**Keywords:** Deep South, COVID-19, vaccine, booster, residential segregation

## Abstract

**Background:**

COVID-19 had a greater impact in the Deep South compared with other regions in the United States. While vaccination remains a top priority for all eligible individuals, data regarding the progress of booster coverage in the Deep South and how the coverage varies by county and age are sparse. Despite existing evidence of racial and ethnic disparities in COVID-19 vaccinations at the individual level, there is an urgent need for evidence at the population level. Such information could highlight vulnerable communities and guide future health care policy-making and resource allocation.

**Objective:**

We aimed to evaluate county-level COVID-19 booster coverage by age group in the Deep South and explore its association with residential segregation.

**Methods:**

An ecological study was conducted at the population level by integrating COVID-19 vaccine surveillance data, residential segregation index, and county-level factors across the 418 counties of 5 Deep South states from December 15, 2021, to October 19, 2022. We analyzed the cumulative percentages of county-level COVID-19 booster uptake by age group (eg, 12 to 17 years, 18 to 64 years, and at least 65 years) by the end of the study period. The longitudinal relationships were examined between residential segregation, the interaction of time and residential segregation, and COVID-19 booster coverage using the Poisson model.

**Results:**

As of October 19, 2022, among the 418 counties, the median of booster uptake was 40% (IQR 37.8%-43%). Compared with older adults (ie, at least 65 years; median 63.1%, IQR 59.5%-66.5%), youth (ie, 12 to 17 years; median 14.1%, IQR 11.3%-17.4%) and adults (ie, 18 to 64 years; median 33.4%, IQR 30.5%-36.5%) had lower percentages of booster uptake. There was geospatial heterogeneity in the county-level COVID-19 booster coverage. We found that higher segregated counties had lower percentages of booster coverage. Such relationships attenuated as time increased. The findings were consistent across the age groups.

**Conclusions:**

The progress of county-level COVID-19 booster coverage in the Deep South was slow and varied by age group. Residential segregation precluded the county-level COVID-19 booster coverage across age groups. Future efforts regarding vaccination strategies should focus on youth and adults. Health care facilities and resources are needed in racial and ethnic minority communities.

## Introduction

Following its approval in September 2021, the booster dose has contributed to more effective prevention and lower mortality for COVID-19 [1,2]. In the United States, the first COVID-19 booster was initially recommended to be administered at least 6 months after the completion of the primary series for individuals aged 18 years and older [2-4]. This guidance later expanded to individuals aged between 16 and 17 years [2-4]. By January 2022, the US Food and Drug Administration [5,6] extended the booster recommendation to those aged 12 to 15 years and shortened the interval to 5 months after the primary vaccination. Despite the encouraging evidence on the efficacy of the COVID-19 vaccine, as of October 19, 2022, only 50.7% of Americans aged 12 years and older had both completed the primary series and received a booster [7,8]. In the Deep South, which includes Alabama, Georgia, Louisiana, Mississippi, and South Carolina, this percentage was even lower (40%-44%) [7,8]. Although around 75% of the individuals aged 12 years and older in the United States (and approximately 63% in the Deep South) are fully vaccinated, the relatively low percentage of booster uptake is concerning as a booster dose can effectively decrease the risk of infection and severe illness [7,8]. Given that the Deep South experienced a disproportionately more severe impact from COVID-19 compared with other regions, vaccination remains a top priority for all eligible individuals [9-11]. However, there are limited data delineating booster coverage in the Deep South and how this rate varied by county and age, which is of critical importance for future vaccine planning, especially for vaccination of updated booster dose.

Racial and ethnic minority communities, including Black and Hispanic communities, are vulnerable to COVID-19 but lack a variety of health care resources. In the prevaccination period of the pandemic, these communities displayed higher incidences of infections, hospitalizations, and unfavorable treatment outcomes [9,12-14]. In the postvaccination period, both Black and Hispanic communities had a lower percentage of completing at least 1 COVID-19 vaccine dose when compared to their White counterparts [15]. This racial and ethnic disparity is also evident among children and adolescents. While the child and adolescent COVID-19 vaccination rates were low overall, Black and Hispanic children and adolescents (aged 5 to 17 years) had lower COVID-19 vaccination rates than their White and Asian peers [16]. One of the major barriers is residential segregation that restricts access to health care resources in minority communities [17]. Empirical research demonstrated that more segregated counties had more pronounced differences in COVID-19 vaccine coverage between Black and White residents [18,19]. However, the vast majority of evidence regarding racial and ethnic disparities in COVID-19 vaccination was generated from the studies at the individual level with limited population-level analyses. Of the few population-based studies, most were cross-sectional designs and did not examine the difference in COVID-19 vaccination by age groups [18,19]. Knowledge gleaned from these age-specific disparities can illuminate the progress of vaccination in racial and ethnic minority communities and facilitate the process of health care policy-making.

Well-designed ecological studies hold the potential to yield compelling evidence on racial and ethnic disparities in vaccination at the population level. Such insights can facilitate the identification of vulnerable communities and guide the optimal allocation of resources for disease control and prevention. A notable strength of ecological studies is their ability to harness comprehensive surveillance data [20,21]. Compared with individual patient data that often require rigorous ethical approvals, surveillance data are publicly accessible. Moreover, by examining racial and ethnic disparities in vaccination at aggregate levels such as communities or counties, ecological studies generate findings that are more generalizable [21]. This is a remarkable strength compared with results generated from analyses that focused on limited individuals and small geographic areas.

In this study, we evaluated the COVID-19 booster coverage by age group among the 418 counties from the 5 Deep South states and examined its relationship with racial and ethnic residential segregation using an ecological design, vaccine surveillance data, and spatiotemporal analysis.

## Methods

### Design, Setting, and Study Period

We conducted an ecological study at the population level by integrating COVID-19 vaccine surveillance data, residential segregation index, and county-level factors from multiple public data sets across the 418 counties of 5 Deep South states (ie, Alabama, Georgia, Louisiana, Mississippi, and South Carolina) from December 15, 2021, to October 19, 2022.

### County-Level COVID-19 Booster Coverage

We retrieved variables regarding vaccine uptake from the Centers for Disease Control and Prevention (CDC) [22] COVID-19 vaccine surveillance data. This data set is representative as it reflects all vaccine partners including jurisdictional partner clinics, retail pharmacies, long-term care facilities, dialysis centers, Federal Emergency Management Agency and Health Resources and Services Administration partner sites, and federal entity facilities. The data regarding the percentage of adults who completed a primary series and have received a booster within a county (henceforth, “county-level booster coverage”) were released on December 15, 2021. The CDC also reported county-level booster coverage for people aged at least 12 years since January 27, 2022. The CDC vaccine surveillance data were updated daily until June 29, 2022, and were then updated weekly.

We retrieved the biweekly cumulative county-level booster uptake for people aged at least 18 years and those aged at least 65 years between December 15, 2021, and October 19, 2022. A total of 23 time points were included in the analyses. Since there was no existing variable for people aged between 18 and 64 years, we manually calculated the biweekly cumulative county-level booster uptake for this group by subtracting the booster uptake of people aged at least 65 years from the overall adults for each county.

For people aged between 12 and 17 years, we retrieved the biweekly cumulative booster uptake for people aged at least 12 years and at least 18 years from January 27 to October 19, 2022. A total of 20 time points were included in the analyses. We used the same method to calculate the biweekly booster uptake for this group in each county.

### Residential Segregation

We defined residential segregation using the index of concentration that measures the relative amount of physical space occupied by minority groups [23,24]. Minority groups of the same relative size occupying less space were considered more concentrated and thus more segregated [23,24]. Since there is a large proportion of the Black population in the Deep South, we considered it as the main minority group and calculated the residential segregation for each county using equation (1):



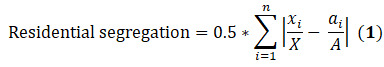



where *n* is the number of tracts in a county, *x_i_* is the size of the Black population in tract *i*, *X* is the size of the Black population in a county, *a_i_* is the land area of tract *i*, and *A* is the total land area in a county [24]. The residential segregation ranged from 0 to 1, with a higher value indicating a higher degree of segregation. In each county, the higher the residential segregation score, the fewer spaces the Black population of the same relative size occupy.

### Potential Confounders

Given the ecological design at the county level, we identified a list of potential county-level confounders based on prevailing literature addressing the structural and social determinants of racial and ethnic disparities of the COVID-19 pandemic in the United States [25]. Thakur et al [25] underscored that racism, social class, and social stratification shaped the risk of exposure to COVID-19 and the access to health care resources through (1) income and occupation, (2) housing and crowding, and (3) health insurance and resource distribution. Therefore, we organized the potential county-level confounders into four dimensions: (1) regional socioeconomic status (ie, the Gini index, the proportion of households with public assistance income [%], the proportion of people in low working class [%], the proportion of people with low education [%], and the proportion of noncitizen [%]); (2) housing and neighborhood environment (ie, household size); (3) health care access and susceptibility (ie, primary care provider rate [per 100,000 people] and proportion of adults who report fair or poor health [%]); and (4) transportation accessibility (ie, proportion of occupied housing units without car access [%]). These variables were validated in prior research to reflect the structural barriers to health care access and delivery [26,27]. We retrieved these variables from multiple public data sets and linked to the county level. Multimedia Appendix 1 shows the definitions and sources of all potential county-level confounders by each dimension.

### Statistical Analysis

Using the geospatial mapping technique, we mapped the cumulative percentages of county-level COVID-19 booster uptake for all ages, people between 12 and 17 years, at least 18 years, between 18 and 64 years, and at least 65 years, respectively. The county-level residential segregation was also mapped. We described the median and IQR for the cumulative percentages of COVID-19 booster coverage at the county level for each of the 5 Deep South states on October 19, 2022.

We used generalized estimating equation with Poisson distribution to examine the relationship between residential segregation and COVID-19 booster coverage rate, adjusting for the repeated measures in each county and potential confounders. Since the US Food and Drug Administration [5,6] advises the first COVID-19 booster dose be administered 5 months after the completion of the primary vaccination series, to generate a robust estimate of the county-level booster coverage rate, we used the 7-day moving average of the total individuals who completed the primary series 5 months before each study time point as an offset in the Poisson model. The model can be presented using equation (2):



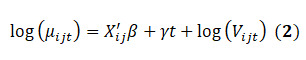



where *µ_ijt_* = *E*(*y_ijt_*|*X_ij_*, *V_ijt_*, *t*) is the marginal mean at time *t* given the covariates, and *y_ijt_* denotes the total number of patients who took a booster at *j*th county within *i*th state during time *t*. The response (county-level COVID-19 booster coverage rate at time *t*) is assumed to be independent across the state but correlated within each county over time. *β* is a vector of regression coefficients, *X_ij_* denotes the vector of county-level variables including residential segregation and other county-level covariates, *γ* denotes the coefficient of time, and *V_ijt_* is the 7-day moving average of the total number of people who completed the primary series of COVID-19 vaccine at county *j* within state *i* at time *t*.

To avoid collinearity, county-level factors were standardized into the same scale with a mean of 0 and an SD of 1 before the analysis. First, we tested the main effects of time and residential segregation on the cumulative percentage of COVID-19 booster coverage. Second, given the temporal trend of COVID-19 vaccination in empirical research, we also examined the interaction between time and residential segregation and sought to understand whether the impact of residential segregation on booster coverage rate changes over time [28]. We used simple slope analysis to interpret interaction [29]. The analysis was replicated for the overall population and by age group. Finally, besides using 5 months as the interval between the primary vaccination series and booster dose as suggested by the US Food and Drug Administration, we did the sensitivity analyses, in which 6-month was used as a cutoff to calculate the offset for the Poisson model. All analyses were conducted using SAS (version 9.4; SAS Institute, Inc).

### Ethical Considerations

The institutional review boards at the University of South Carolina approved the study protocol (PRO00100854). This study was an ecological analysis based on the CDC vaccine surveillance data and multiple public access data sets. No personal identification information was involved in this analysis.

## Results

### Overview

A total of 418 counties across 5 Deep South states were included in this study. There were 67, 159, 64, 82, and 46 counties in Alabama, Georgia, Louisiana, Mississippi, and South Carolina, respectively.

As of October 19, 2022, among the 418 counties, the median of booster uptake was 40% (IQR 37.8%-43%). In the individual states, the median of county-level booster uptake ranged from 38.4% (IQR 36.2%-40.1%) in Alabama to 43.4% (IQR 40.5%-45.4%) in South Carolina (Table 1).

**Table 1:** Cumulative percentages of county-level COVID-19 booster uptake for the overall sample and by age group across the 5 Deep South states, as of October 19, 2022.

States and age groups				Values (%), median (IQR)
**Deep South (N=418)**				
	Overall population			40 (37.8-43)
	12 to 17 years^a^			14.1 (11.3-17.4)
	**Overall adults**			
		≥18 years	42.2 (39.8-45.1)	
		18 to 64 years	33.4 (30.5-36.5)	
		At least 65 years	63.1 (59.5-66.5)	
**Alabama (n=67)**				
	Overall population			38.4 (36.2-40.1)
	12 to 17 years^a^			13 (10.5-15.6)
	**Overall adults**			
		≥18 years	40 (37.8-41.8)	
		18 to 64 years	30.2 (28.1-33.8)	
		At least 65 years	59.5 (57.5-61.7)	
**Georgia (n=159)**				
	Overall population			40.2 (37.5-43.2)
	12 to 17 years^a^			14.9 (11.8-17.8)
	**Overall adults**			
		≥18 years	42 (39.6-45.4)	
		18 to 64 years	33.8 (31.2-36.6)	
		At least 65 years	61.6 (58.3-64.8)	
**Louisiana (n=64)**				
	Overall population			40.7 (38.5-43.6)
	12 to 17 years^a^			13.5 (11.2-16.8)
	**Overall adults**			
		≥18 years	42.7 (40.5-45.9)	
		18 to 64 years	33.3 (30.3-36.1)	
		At least 65 years	67.4 (63.4-71.8)	
**Mississippi (n=82)**				
	Overall population			39.9 (37.7-41.1)
	12 to 17 years^a^			12.6 (10.6-16)
	**Overall adults**			
		≥18 years	42.2 (39.9-44.1)	
		18 to 64 years	32.5 (30.4-35.7)	
		At least 65 years	64 (61.1-67.3)	
**South Carolina (n=46)**				
	Overall population			43.4 (40.5-45.4)
	12 to 17 years^a^			16.9 (14-20.3)
	**Overall adults**			
		≥18 years	46 (43.2-47.9)	
		18 to 64 years	36.2 (33.9-37.6)	
		At least 65 years	66.4 (64.4-69.4)	

^a^The first record for the group of 12 to 17 years was available on January 27, 2022. For other groups, the first record was available on December 15, 2021.

The percentage of booster uptake for people aged at least 65 years was high with a median value of 63.1% (IQR 59.5%-66.5%) across the 418 counties. The percentage of booster uptake for people aged between 12 and 17 years was low with a median value of 14.1% (IQR 11.3%-17.4%). For people aged between 18 and 64 years, the percentage of booster coverage was 33.4% (IQR 30.5%-36.5%). Table 1 depicts the cumulative county-level percentage of COVID-19 booster uptake by age group across the 5 Deep South states, as of October 19, 2022.

### Geospatial Heterogeneities in Residential Segregation and County-Level Booster Coverage

**Figure 1:**
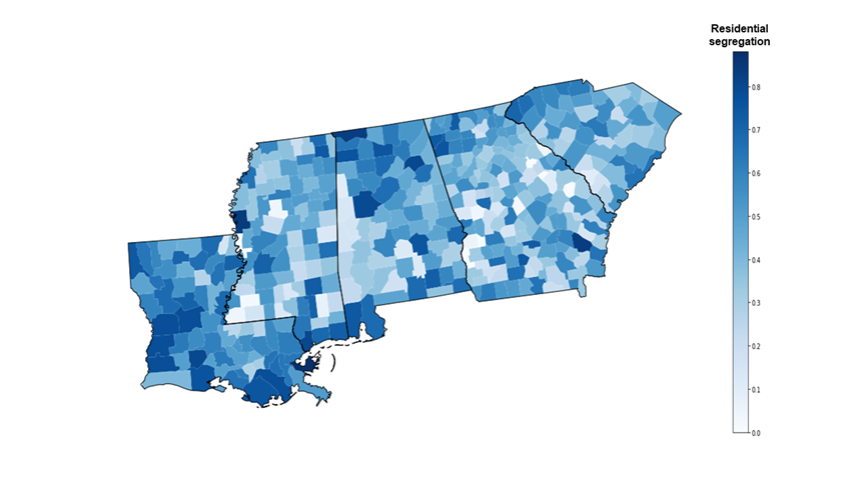
County-level residential segregation in the 5 Deep South states. From left to right, the states were Louisiana, Mississippi, Alabama, Georgia, and South Carolina, respectively.

**Figure 2:**
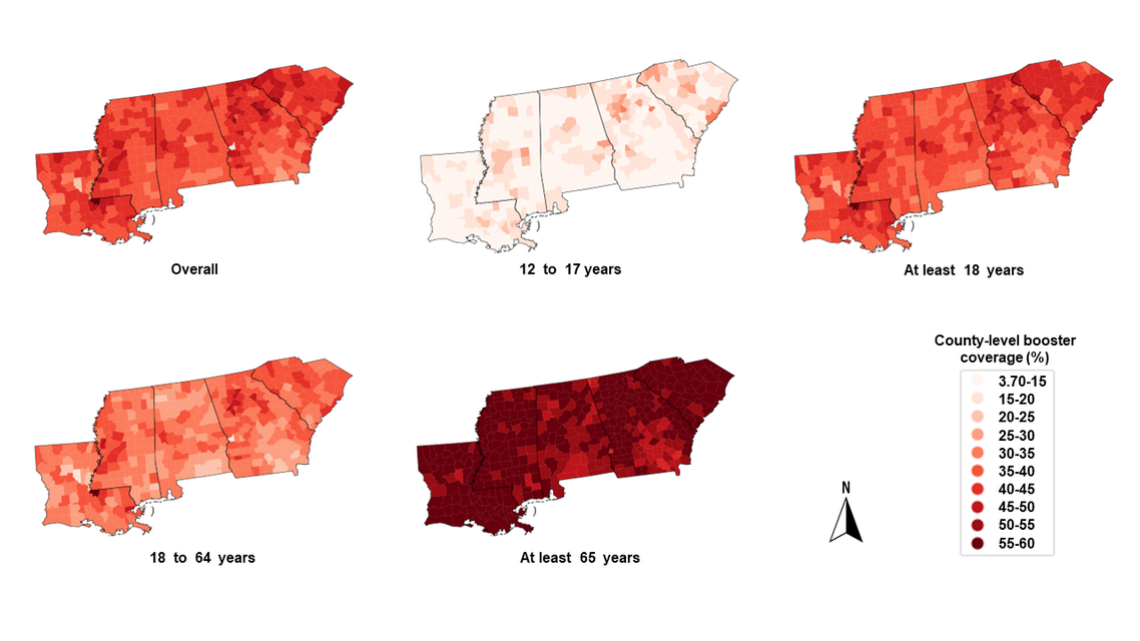
County-level COVID-19 booster coverage for the overall sample and by age group in the 5 Deep South states on October 19, 2022. From left to right, the states were Louisiana, Mississippi, Alabama, Georgia, and South Carolina, respectively.

Figures 1 and 2 show the distribution of county-level residential segregation score and booster uptake, respectively. In Figure 1, counties in dark blue had higher residential segregation scores than those in light blue. There are some geospatial clusters with high levels of residential segregation within each state. For instance, Louisiana had more counties in dark blue than the other 4 states, indicating that counties in Louisiana were more segregated. These counties had high levels of residential segregation and were mainly located in the southwestern areas. In South Carolina, counties located in the northwestern and southeastern regions are more segregated compared with others.

In Figure 2, counties in dark red had higher percentages of booster uptake than those in light red. In general, the percentage of completing primary series with 1 booster among people aged at least 65 years was higher than that in other age groups. In each individual state, there were some counties with low booster uptake. These counties were mainly located in southern Alabama, southeastern Georgia, southwestern and central Louisiana, southwestern and central Mississippi, and northern South Carolina. Figure 2 also shows the county-level booster uptake for other age groups.

Taken Figures 1 and 2 together, there were some counties in which there was high residential segregation but low booster uptake. Some of these counties were Franklin and Coffee in Alabama, Bacon and Colquitt in Georgia, Vernon and Acadia in Louisiana, Hancock and Lauderdale in Mississippi, and Cherokee and Dorchester in South Carolina.

### Relationship Between Residential Segregation and County-Level Booster Coverage

In general, as time increased, the change rate of county-level booster coverage decreased for people aged at least 18 years (β=–.051. 95% CI –0.072 to –0.031), between 18 and 64 years (β=–.053, 95% CI –0.079 to –0.027), and at least 65 years (β=–.028, 95% CI –0.044 to –0.012). For people aged between 12 and 17 years, with time increased, the change rate increased (β=.055, 95% CI 0.037 to 0.074).

Residential segregation was negatively associated with the county-level booster coverage in people aged at least 18 years (β=–.067, 95% CI –0.088 to –0.047), between 18 and 64 years (β=–.074, 95% CI –0.099 to –0.049), and at least 65 years (β=–.044, 95% CI –0.060 to –0.028). However, this negative association was not found in the analysis in people aged between 12 and 17 years (β=–.009, 95% CI –0.039 to 0.020).

**Figure 3:**
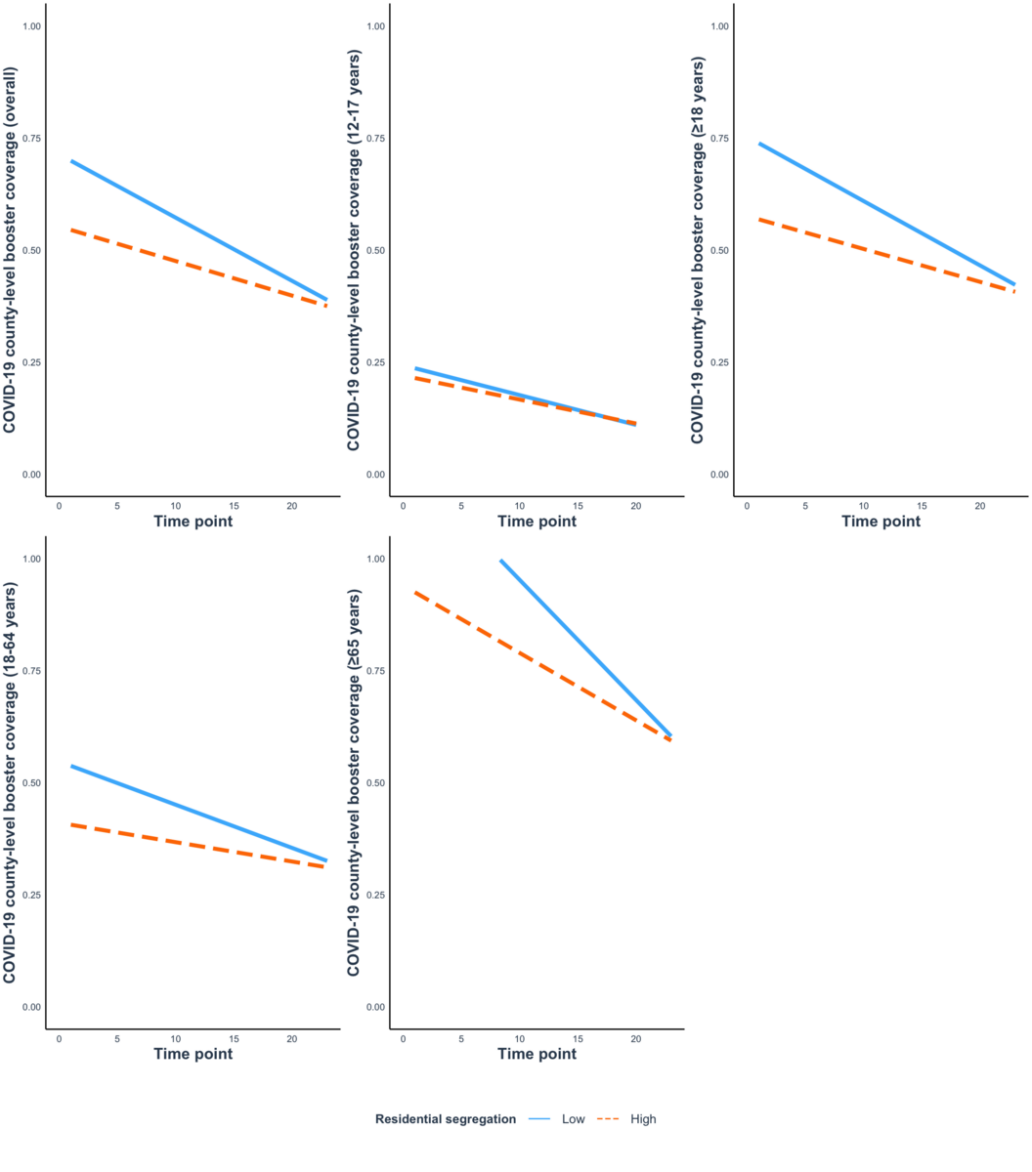
Simple slope plots for interaction between time and residential segregation for the overall sample and by age group.

In the analyses that examined the interaction between time and residential segregation on the county-level booster coverage, we consistently found a significant interaction for the overall sample (β=.054, 95% CI 0.040 to 0.069) and by age group (between 12 and 17 years: β=.031, 95% CI 0.018 to 0.044; at least 18 years: β=.057, 95% CI 0.042 to 0.072; between 18 and 64 years: β=.065, 95% CI 0.049 to 0.082; and at least 65 years: β=.047, 95% CI 0.032 to 0.062; Table 2). Simple slope analyses indicated that at a given time point, counties characterized by residential segregation above the mean (high residential segregation) experienced lower COVID-19 county-level booster coverage compared to counties where residential segregation was at or below the mean (low residential segregation). However, such difference attenuated as time increased (Figure 3).

**Table 2:** Residential segregation and COVID-19 booster coverage for the overall sample and by age group in the 418 counties across the 5 Deep South states from December 15, 2021, to October 19, 2022^a^.

Model and variable		Overall^b^, β (95% CI)		12-17 years^c^, β (95% CI)		≥18 years, β (95% CI)		18-64 years, β (95% CI)		≥65 years, β (95% CI)
**Model 1: main effects^d^**										
	Time	–.058 (–0.078 to –0.038)^e^	.055 (0.037 to 0.074)^e^		–.051 (–0.072 to –0.031)^e^		–.053 (–0.079 to –0.027)^e^		–.028 (–0.044 to –0.012)^e^	
	Residential segregation	–.066 (–0.086 to –0.045)^e^	–.009 (–0.039 to 0.020)		–.067 (–0.088 to –0.047)^e^		–.074 (–0.099 to –0.049)^e^		–.044 (–0.060 to –0.028)^e^	
**Model 2: main effects and interaction^d^**										
	Time	–.082 (–0.100 to –0.064)^e^	.044 (0.026 to 0.061)^e^		–.077 (–0.095 to –0.058)^e^		–.080 (–0.102 to –0.058)^e^		–.051 (–0.068 to –0.034)^e^	
	Residential segregation	–.073 (–0.094 to –0.052)^e^	–.016 (–0.046 to 0.014)		–.075 (–0.096 to –0.053)^e^		–.083 (–0.109 to –0.058)^e^		–.049 (–0.065 to –0.032)^e^	
	Time×residential segregation	.054 (0.040 to 0.069)^e^	.031 (0.018 to 0.044)^e^		.057 (0.042 to 0.072)^e^		.065 (0.049 to 0.082)^e^		.047 (0.032 to 0.062)^e^	

^a^Unless otherwise noted, the study period was from December 15, 2021, to October 19, 2022.

^b^From December 15, 2021 to January 26, 2022, the overall population referred to people aged at least 18 years. Since January 27, 2022, it referred to people aged at least 12 years.

^c^The first record for the group of 12 to 17 years was available on January 27, 2022. For other groups, the first record was available on December 15, 2021.

^d^Confounders: Gini index, proportion of households with public assistance income, proportion of people in low working class, proportion of people with low education, proportion of noncitizen, household size, primary care provider rate, proportion of adults who report fair or poor health, proportion of occupied housing units without access to a vehicle.

^e^*P*≤.05.

The sensitivity analyses using 6 months as a cutoff to derive the offset in Poisson model found that the regression coefficients for the overall population and specific age groups closely aligned with those from the primary analyses (Multimedia Appendix 2). Residential segregation was not significantly associated with county-level COVID-19 booster coverage in the subgroup analysis for people aged 12 to 17 years. The sensitivity analyses corroborated our main findings and did not change the interpretation.

## Discussion

### Principal Findings

In a longitudinal analysis across the 418 counties in the Deep South, we found county-level COVID-19 booster coverage was generally low and exhibited variations across different age groups. Compared with the older adult population, booster uptake was lower in youth (ie, 12 to 17 years) and adults (ie, 18 to 64 years). There was a geospatial heterogeneity in the county-level COVID-19 booster coverage, which was negatively associated with residential segregation. Specifically, counties with higher levels of segregation experienced lower booster coverage. However, this disparity decreased over time. This study was innovative as we evaluated the county-level booster coverage by age group from a longitudinal perspective, which was rare in previous research. Our findings were in line with those from the analyses at the individual level and CDC reports, which found that vaccination varied by age and racial and ethnic groups [8,22,30].

The progress of county-level COVID-19 booster coverage in the Deep South was slow and insufficient to curb the transmission of new COVID-19 variants from person to person. This slow progress was found in youth and adults. While the slow progress in these 2 age groups might be due to the low percentage of people who were fully vaccinated and eligible for a booster dose, it might also be due to the fear of long-term effects and serious side effects among the parents and the adults themselves [31,32]. Additionally, some young adults hesitated to get vaccinated against COVID-19 because they did not think it is necessary or beneficial [31,32]. Denford et al [31] found that people were unvaccinated because they considered themselves to be young, healthy, and at low risk of getting sick. However, to effectively control the pandemic in the United States, at least 70% of the population needs to get vaccinated although it is challenging.

Our findings indicated that residential segregation reflects racial and ethnic disparities at the county level and negatively impacts on the COVID-19 booster coverage, which was consistent across all age groups. Defined as the relative amount of physical space occupied by the Black population, the residential segregation in this study had consistent distributions with data from the National Institute on Minority Health and Health Disparities, demonstrating the validity of our residential segregation index [33]. There are more and more research conducted at the population level that examine the association of residential segregation with health outcomes [18,19]. Our findings add value to the existing literature by reinforcing the validity of county-level residential segregation as an indicator of racial and ethnic disparities. The negative impact of residential segregation on COVID-19 booster coverage was consistent with the empirical findings [17-19]. Racial and ethnic minority communities often experienced a scarcity of vaccine distribution sites and vaccine doses, resulting in low vaccination rates in these areas [17]. Additionally, the unequal distribution of educational resources and opportunities in highly segregated communities may preclude health education regarding the safety and effectiveness of the COVID-19 vaccine and serve as a roadblock to county-level booster coverage [34].

Our findings provide critical insights into the design of vaccine surveillance studies and the development of public health interventions to enhance booster coverage. From the research perspective, our findings demonstrated the strength of ecological study and confirmed that well-designed ecological research can yield consistent results with analyses based on individual patient data. Importantly, our conclusions had strong external validity and can be generalized to racial and ethnic minority communities in other parts of the United States. From a public health perspective, there is a pressing need to enhance vaccination initiatives in the Deep South, with a particular focus on youth, parents, and adults. Community-based health education campaigns should emphasize the safety and effectiveness of the COVID-19 vaccine. Given the disparities in health care access within racial and ethnic minority communities, to promote booster coverage, there is an urgent need to prioritize the allocation of health care facilities and resources in these areas.

### Limitations

There are several limitations in this study. First, this was an ecological study focusing on county-level COVID-19 booster coverage and its contextual factors. Our findings might suffer from the ecological fallacy [20]. Caution may be needed when interpreting our findings at the individual level. Second, residential segregation was calculated based on the Black population, given the large proportion of the Black population in the Deep South. Consequently, our findings only reveal the relationship between residential segregation and booster coverage in Black communities. Third, although our analysis adjusted for a list of important county-level confounders that were selected based on a strong conceptual model and validated in prior research, other unadjusted confounders, such as political ideology and religious culture, might attenuate or enlarge the effect of residential segregation on booster coverage [15,25]. Our findings may be affected by residual confounding. We recommended future studies examine our findings with the consideration of more validated confounders. Fourth, given the nature of ecological design at the county level, we did not include individual factors in this study. Future studies incorporating both county- and individual-level factors can explore the interactions between them in predicting booster coverage. Finally, we used a proxy number of people who completed the primary series 5-month before each selected time point as an offset to model the booster coverage rate in the Poisson model, since this study was based on vaccine surveillance data, and no personal identifiers were involved. We did not have the information regarding vaccine types (eg, Pfizer-BioNTech, Moderna, and Johnson & Johnson) and the actual time interval between primary series and booster for each individual, which precluded us to model booster coverage uptake precisely. To counteract this limitation, we used the 7-day moving average of individuals who completed the primary series in our analyses. We also did sensitivity analyses using 6 months as an interval. The results were consistent with those from the analyses using 5 months. We suggest that future studies use health care administrative data to further validate our findings.

### Conclusions

The progress of county-level COVID-19 booster coverage in the Deep South was slow and varied by age group. The progress was even slower in youths and adults as compared with older adults. The residential segregation precluded booster coverage across the age groups. Future efforts regarding vaccine planning and implementation should focus on the youths and adults. Health care facilities and resources are needed in racial and ethnic minority communities.

## Appendix

Multimedia Appendix 1 Definitions and sources of the proposed county-level confounders by each dimension.

Multimedia Appendix 2 Residential segregation and COVID-19 booster coverage by age group in the 418 counties across the 5 Deep South states from December 15, 2021, to October 19, 2022.

## Abbreviations

CDC: Centers for Disease Control and Prevention

## Data Availability

Vaccine uptake data and county-level factors are included in this published paper. Residential segregation score is available from the corresponding author on reasonable request.
