# Supplementary material for: Residential Segregation and County-Level COVID-19 Booster Coverage in the Deep South: Surveillance Report and Ecological Study
Source: JMIR Public Health Surveill. 2023 Dec 5;9:e44257. doi: 10.2196/44257 (PMC10699407; doi:10.2196/44257)
Supplement: Multimedia Appendix 1 [file publichealth_v9i1e44257_app1.docx]

**Multimedia Appendix 1.** Definitions and sources of the proposed county-level confounders by each dimension.

| **Proposed confounders** | **Definitions** | **Sources** |
| --- | --- | --- |
| **Demographic characteristics** |  |  |
| Gini index | A measure of statistical dispersion represents the income inequality or wealth inequality within a group of people at local areas. It ranges from 0 (perfect equality) to 1 (perfect inequality). | American Community Survey |
| Public assistance | Proportion of households with public assistance income |  |
| Low working class | Proportion of people in specific occupations, such as service occupations, sales and office occupations, farming, fishing and forestry occupations, construction, extraction and maintenance occupations, production transportation and material moving occupations |  |
| Low education | Proportion of people aged 25 years and older, with less than 12^th^ grade education |  |
| Noncitizen | Proportion of noncitizen |  |
| **Housing and neighborhood environment** | | |
| Household size | Proportion of occupied housing units with more than one occupant per room | American Community Survey |
| **Healthcare access and susceptibility** | |  |
| Primary care provider rate | Numbers of primary care provider per 100,000 people | U.S. Health Data |
| Adults that report fair or poor health | Proportion of adults who report fair or poor health status |  |
| **Transportation accessibility** |  |  |
| Car access | Proportion of occupied housing units without access to a vehicle | American Community Survey |
