# Supplementary material for: Residential Segregation and County-Level COVID-19 Booster Coverage in the Deep South: Surveillance Report and Ecological Study
Source: JMIR Public Health Surveill. 2023 Dec 5;9:e44257. doi: 10.2196/44257 (PMC10699407; doi:10.2196/44257)
Supplement: Multimedia Appendix 2 [file publichealth_v9i1e44257_app2.docx]

**Multimedia Appendix 2.** Residential segregation and COVID-19 booster coverage by age group in the 418 counties across the 5 Deep South states from December 15, 2021, to October 19, 2022.^∆,†^

| **Model 1** | **Main effects** | | | | |
| --- | --- | --- | --- | --- | --- |
|  | **Overall^ǁ^** | **12-17 years^‡^** | **≥ 18 years** | **18-64 years** | **≥ 65 years** |
| Time | -0.084  (-0.103, -0.066)* | -0.025  (-0.040, -0.010)* | -0.074  (-0.094, -0.055)* | -0.083  (-0.106, -0.059)* | -0.035  (-0.050, -0.019)* |
| Residential segregation | -0.073  (-0.096, -0.050)* | -0.013  (-0.041, 0.016) | -0.075  (-0.098, -0.052)* | -0.082  (-0.109, -0.055)* | -0.051  (-0.070, -0.032)* |
| **Model 2** | **Main effects and interaction** | | | | |
| Time | -0.107  (-0.124, -0.090)* | -0.033  (-0.048, -0.019)* | -0.097  (-0.115, -0.079)* | -0.107  (-0.127, -0.088)* | -0.055  (-0.072, -0.039)* |
| Residential segregation | -0.080  (-0.104, -0.056)* | -0.018  (-0.047, 0.011) | -0.082  (-0.106, -0.058)* | -0.091  (-0.119, -0.064)* | -0.055  (-0.075, -0.036)* |
| Time × residential segregation | 0.051  (0.037, 0.066)* | 0.022  (0.009, 0.035)* | 0.053  (0.038, 0.068)* | 0.061  (0.045, 0.076)* | 0.043  (0.028, 0.058)* |

∆: 6-month was used as a cutoff to calculate the offset for the Poisson model.

†: Unless otherwise noted, study period was from December 15, 2021 to October 19, 2022.

‡: The first record for the group of 12 to 17 years old was available on January 27, 2022. For other groups, the first record was available on December 15, 2021.

ǁ: From December 15, 2021 to January 26, 2022, the overall population referred to people at least 18 years old. Since January 27, 2022, it referred to people at least 12 years old.

Confounders: Gini index, proportion of households with public assistance income, proportion of people in low working class, proportion of people with low education, proportion of noncitizen, household size, primary care provider rate, proportion of adults who report fair or poor health, proportion of occupied housing units without access to a vehicle.

*: *p* ≤ 0.05.
